# The Role of Multidimensional Indices for Mortality Prediction in Chronic Obstructive Pulmonary Disease

**DOI:** 10.3390/diagnostics13071344

**Published:** 2023-04-04

**Authors:** Stanislav Kotlyarov

**Affiliations:** Department of Nursing, Ryazan State Medical University, 390026 Ryazan, Russia; skmr1@yandex.ru

**Keywords:** COPD, survival, prognosis, CODEX, eBODE, BODE, ADO, BODEX, Charlson Comorbidity Index, COTE

## Abstract

(1) Background: Chronic obstructive pulmonary disease (COPD) is one of the most important respiratory diseases. It is characterised by a progressive course with individual differences in clinical presentation and prognosis. The use of multidimensional indices such as the BODE, eBODE, BODEX, CODEX, ADO, and Charlson Comorbidity Index has been proposed to predict the survival rate of COPD patients. However, there is limited research on the prognostic significance of these indices in predicting long-term survival rates in patients with COPD. The aim of this prospective cohort study was to investigate the prognostic value of the BODE, eBODE, BODEX, CODEX, ADO, COTE and Charlson Comorbidity Index in predicting 5- and 10-year survival in patients with COPD. (2) Methods: A total of 170 patients were included in the study and their clinical and functional characteristics of COPD progression, such as dyspnoea, body mass index and spirometry data, were evaluated. A Kaplan–Meier survival analysis was used to calculate 5- and 10-year survival rates. The predictive value of each index was assessed using Cox proportional hazards regression models. (3) Results: The 5-year survival rate was 62.35% and the 10-year survival rate was 34.70%. The BODE, eBODE, BODEX, CODEX, ADO, COTE and Charlson Comorbidity Index were all significantly associated with the 10-year survival rate of COPD patients (*p* < 0.05). The hazard ratios (HRs) for these indices were as follows: BODE (HR = 1.30, 95% confidence interval [CI] 1.21–1.39); eBODE (HR = 1.29, 95% CI 1.21–1.37); BODEX (HR = 1.48, 95% CI 1.35–1.63); CODEX (HR = 1.42, 95% CI 1.31–1.54); COTE (HR = 1.55, 95% CI 1.36–1.75); ADO (HR = 1.41, 95% CI 1.29–1.54); and Charlson Comorbidity Index (HR = 1.35, 95% CI 1.22–1.48). (4) Conclusions: The multidimensional indices are a useful clinical tool for assessing the course and prognosis of COPD. These indices can be used to identify patients at a high risk of mortality and guide the management of COPD patients.

## 1. Introduction

Chronic obstructive pulmonary disease (COPD) is an important medical and social problem due to its high prevalence, impact on quality of life, contribution to hospitalisation and temporary and permanent disability [1]. In addition, COPD is one of the leading causes of death worldwide. The disease places a heavy economic and social burden on patients, societies and healthcare systems in many countries. These and other data have increased the focus on early diagnosis and effective treatment of patients with COPD.

Smoking is thought to be a major cause of COPD, contributing to oxidative stress and chronic inflammation in the airways [2,3,4,5]. Air pollutants characteristic of industrial pollution in large cities also contribute to the development of the disease [6].

Chronic inflammation develops over many years, leading to airway obstruction and respiratory symptoms such as chronic cough, sputum production and breathlessness. However, many smokers may not seek medical help for a long time, believing that these symptoms are a natural manifestation of smoking. In addition, many patients do not stop smoking even after they have been diagnosed with COPD. Patients’ adherence to treatment also remains unresolved, giving a generally negative picture of the prevalence of COPD and its contribution to disability and mortality. Assessing the course and prognosis of COPD is, therefore, of particular clinical interest.

In the complex history of research into this problem, various characteristics of the course of COPD have been proposed. Pulmonary function testing is an important diagnostic tool that underpins the classification of COPD according to severity. However, it has been shown that simple spirometric classification does not fully reflect the course and prognosis of COPD and that other more accurate markers are needed [6]. The evaluation of markers of COPD progression and prognosis is of great clinical interest. COPD is known to have pulmonary and extrapulmonary clinical heterogeneity, manifested by variability in clinical features such as severity of symptoms, dynamics of pulmonary function decline, and presence and type of comorbidities. The clinical heterogeneity of COPD phenotypes prevents the development of a single tool for predicting disease progression and clinical outcomes, so multivariate indices assessing different clinical features of the disease have been developed. Multidimensional indices were better predictors of survival than either index alone. Multidimensional indices have been found to have good prognostic value and can be used to assess the course and survival of COPD in long-term follow-up. Several indices are widely accepted, such as the BODE index and its modifications for exacerbation frequency (BODEX and eBODE), CODEX, ADO, and tools for prognostically significant comorbidity, such as the Charlson Comorbidity Index and COTE [7,8]. They differ in the variables analysed, but all incorporate available clinical data and are easy to use in real clinical practice [9].

The BODE multidimensional index incorporates the following measures: BMI (B); airflow obstruction (O); dyspnoea, as measured by the modified Medical Research Council (mMRC) scale (D); and exercise intolerance (E), as measured by the six-minute walk distance (6MWD) [8]. The multidimensional BODE index has shown good prognostic value in many studies of COPD. However, the BODE index does not take into account the frequency of COPD exacerbations, which plays an important role in the natural history of COPD. Modifications of the BODE index have been proposed, such as the BODEX index (BMI, airflow obstruction, dyspnoea, exacerbation) [10] and e-BODE (exacerbation and BODE) [10]. These indices have shown higher prognostic value in patients with COPD.

The multidimensional ADO index includes an assessment of age (Age—A), dyspnoea severity (Dyspnoea—D) and FEV1 (Obstruction—O) [11]. The ADO index is of considerable practical interest because of its simplicity and ease of calculation.

The Charlson Comorbidity Index was proposed in 1987 to assess comorbid conditions. The index is a system for scoring the presence of certain comorbidities according to the age of the patient. A certain number of points are assigned to comorbidities, and 1 point is added for each decade of age if the patient is over 40 years of age. The CODEX index is another tool to assess the impact of comorbidity on prognosis. In addition to comorbidity (based on the Charlson Comorbidity Index Scale), this multidimensional index also includes the degree of airflow obstruction, the severity of dyspnoea and the presence of COPD exacerbations [12].

Numerous studies have shown varying degrees of effectiveness in assessing long-term prognosis. Knowing the prognosis of the disease and the factors that predict an adverse outcome in COPD is clinically important because it allows clinicians to predict the expected natural history of the disease and the likelihood of complications. It is also important for making decisions about disease therapy or the intensity of disease monitoring.

Therefore, the aim of the present study was to evaluate the significance of prognostic tools in chronic obstructive pulmonary disease.

## 2. Materials and Methods

### 2.1. Study Design

This was a prospective cohort study designed to evaluate the prognostic value of multidimensional indices in male patients with COPD. The study was conducted in the Ryazan region of Russia from November 2007 to November 2022.

**Figure 1:**
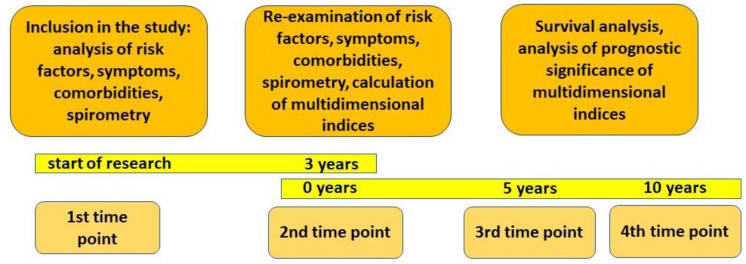
Scheme of the study design.

The study design included an analysis of data at four time control points (Figure 1).

**Figure 2:**
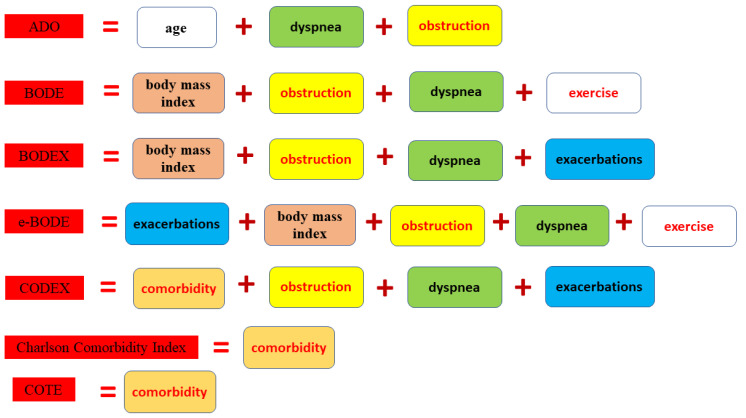
Multidimensional indices analysed in this study.

The first control point was when the patient was enrolled in the study. This consisted of a review of the patient’s medical history, including risk factors and medical records. All patients had a clinical examination and spirometry. The second control point was 3 years after the first. It included a reassessment of risk factors, clinical examination of patients, spirometry, and analysis of medical records. Data on the progression of COPD between the first and second control points made it possible to assess the individual nature of the progression of COPD. The clinical data obtained at this time point were used to calculate the multidimensional indices BODE, BODEX, e-BODE, ADO, CODEX, COTE, and Charlson comorbidity index (Figure 2). The third and fourth control points were 5 and 10 years after the second control point, respectively. At this stage, medical records were analysed, including information on the cause of death, and the patients’ 5-year and 10-year survival rates after the second control point were assessed.

### 2.2. Study Participants

A total of 170 male patients with COPD were enrolled. Inclusion criteria were a confirmed diagnosis of COPD based on the Global Initiative for Chronic Obstructive Lung Disease—GOLD criteria and voluntary informed consent to participate in the study. Exclusion criteria: (1) the presence of any other chronic respiratory disease, including a history of asthma, or an increase in FEV1 greater than 15% or more than 200 mL from baseline after inhaled salbutamol; (2) known cancer of any site, HIV infection and other immunodeficiencies, mental disorders and other conditions manifested by an inability to understand and comply with the protocol, and no long-term follow-up due to severity of clinical condition.

The clinical examination of the patients included an analysis of chronic respiratory symptoms, frequency of COPD exacerbations, body mass index, 6MWD and identification of comorbidities. Dyspnoea severity was assessed using the modified Medical Research Council Dyspnea Scale (mMRC).

All patients underwent spirometry twice: the first time at baseline and a second time 3 years later. COPD exacerbations were diagnosed according to the criteria of N.R. Anthonisen et al. [13]. To objectify the data, the mean frequency of exacerbations per year was calculated, taking into account the three-year data between the first and second control points.

The clinical characteristics of the patients included in the study are shown in Table 1.

### 2.3. Data Analysis and Statistical Processing

The prognostic significance of the multivariate indices was assessed by a Kaplan–Meier analysis of long-term survival. The statistical significance of differences between the curves was assessed using the LogRank (Mantel–Cox) test. Comparison results were considered statistically significant at *p* < 0.05. The predictive value of each index was assessed using Cox proportional hazards regression models. The ability of the BODE, BODEX, e-BODE, ADO, CODEX, COTE and Charlson comorbidity indices to predict mortality was compared using receiver operating characteristic (ROC) curves.

Data were statistically processed using MedCalc 20.1.4 and R software (version 4.2.2). Data are presented with 95% confidence intervals (CI) around the mean. Categorical data were compared between subgroups using the chi-squared test, and continuous variables were compared using Student’s *t*-test or Mann–Whitney U-test, analysis of variance (ANOVA), or Kruskal–Wallis ANOVA after evaluation of the criteria using parametric tests. Differences meeting the *p* < 0.05 criterion were considered statistically significant. Data were visualised using http://www.bioinformatics.com.cn (accessed on 1 March 2023).

### 2.4. Ethical Approval

The protocol of this study was approved by the Ethical Committee of the Ryazan State Medical University (protocol No. 2, 12 November 2007).

## 3. Results

A total of 170 patients with COPD were included in the study. All patients were smokers at baseline and the mean pack-year index was 37.72 (95% CI 36.41, 39.03).

The 5-year survival rate from the second control point was 62.35% and the 10-year survival rate was 34.70%. Causes of death were COPD in 10 patients (9%), cancer in 10 patients (9%), cerebrovascular disease in 46 patients (41.45%), cardiovascular disease and PAD in 31 patients (27.94%), and other causes including trauma, cirrhosis and others in 14 patients (12.61%).

**Figure 3:**
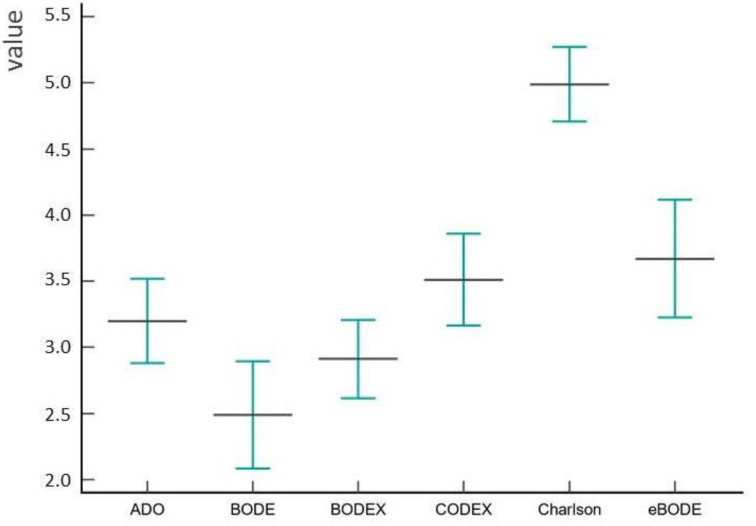
Diagram of the index values at the second control point.

The BODE, BODEX, e-BODE, ADO, Charlson Comorbidity Index, COTE and CODEX index values calculated at the second control point are shown in Figure 3.

The analysis showed the association of the indices with the COPD stage, among which the BODEX, eBODE, CODEX, and BODE had the best correlations, whereas the Charlson Comorbidity Index and the COTE index had the least association with COPD stage (Table 2).

**Figure 4:**
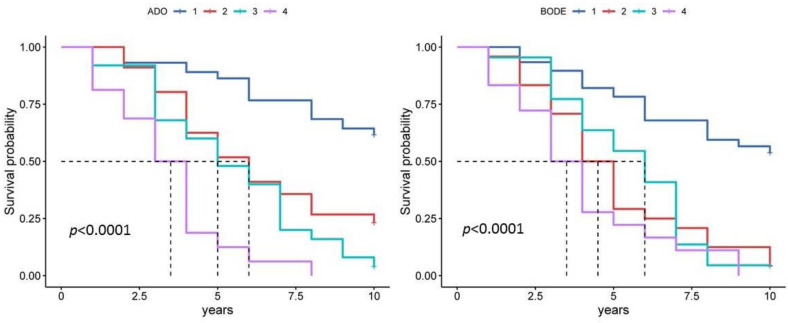
Kaplan–Meier curve plot for ADO and BODE index in estimating 10-year survival. Note: Quartile 1 is a score of 0 to 2, quartile 2 is a score of 3 to 4, quartile 3 is a score of 5 to 6 and quartile 4 is a score of 7 to 10.

The analysis showed some differences in the prognostic significance of the multidimensional indices. The ADO index had good prognostic significance for both 5-year and 10-year survival (Figure 4, Table 3 and Table 4). ADO index values range from 0 to 10 points, with 10 representing the maximum risk of death. Patients were categorized into low-risk (ADO score 0–2), intermediate-risk (ADO score 3–4), high-risk (ADO score 5–7) and very high-risk (ADO score 8–10) groups. The median ADO index score was 3.2, 95% CI [2.88, 3.52] (Figure 3). The ADO index was significantly associated with all-cause mortality (*p* < 0.001). A Kaplan–Meier curve plot considering ADO index values divided into quartiles showed that patients with the fourth quartile had a worse prognosis compared to the first quartile (HR 7.76, 95% CI [2.98, 20.20], *p* < 0.001). (Figure 4). An ROC analysis showed that the ADO index had good predictive accuracy for 5-year mortality (AUC = 0.791, 95% CI [0.722–0.849], *p* < 0.001) and 10-year mortality (AUC = 0.826, 95% CI [0.760–0.880], *p* < 0.001). Our study showed that the ADO index is a useful tool for assessing prognosis in patients with COPD. Patients with higher ADO scores have an increased risk of mortality. The ADO index is easy to calculate in clinical practice and can be used as an adjunct to lung function tests in predicting outcome in patients with COPD.

The BODE index, the best-known multidimensional index, also showed good predictive value. Like the ADO index, the BODE index values range from 0 to 10 points. The median BODE index score was 2.49, 95% CI [2.08, 2.89] (Figure 3). A Kaplan–Meier curve plot considering BODE index values divided into quartiles showed that patients with the fourth quartile had a worse prognosis compared to the first quartile (HR 5.03, 95% CI [2.20, 11.49], *p* < 0.001) (Figure 4). Survival was significantly different between groups (*p* < 0.0001 by log-rank test). The ROC analysis confirmed its prognostic significance for both 5-year and 10-year survival (Table 3 and Table 4). In contrast to the ADO index, the BODE index does not take age into account, but rather the results of the 6MWD test, which can be difficult in real clinical practice. The BODE index also includes body mass index. A body mass index < 21 kg/m^2^ is known to be an independent prognostic factor for long-term mortality.

**Figure 5:**
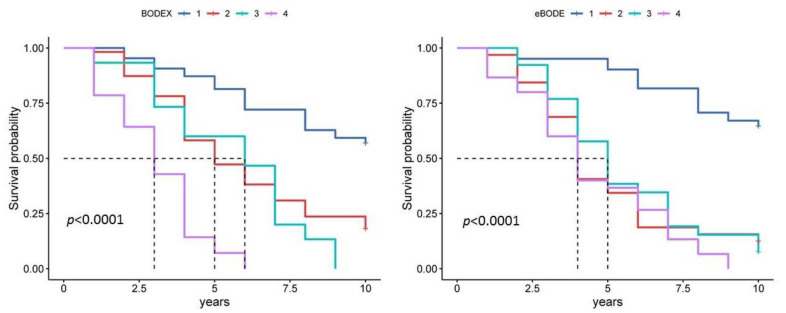
Kaplan–Meier curve plot for the BODEX index and the eBODE index in estimating 10-year survival. Notes: For the BODEX index, quartile 1 is a score of 0 to 2, quartile 2 is a score of 3 to 4, quartile 3 is a score of 5 to 6 and quartile 4 is a score of 7 to 9. For the eBODE index, quartile 1 is a score of 0 to 2, quartile 2 is a score of 3 to 4, quartile 3 is a score of 5 to 6 and quartile 4 is a score of 7 to 12.

The BODEX index, which unlike the BODE includes body mass index (B), degree of airflow obstruction (O), dyspnoea severity according to the mMRC scale (D) and exacerbation frequency (EX), also showed good prognostic significance (Figure 5, Table 3 and Table 4). The inclusion of exacerbation frequency in the BODEX index increased its prognostic significance compared to BODE (Table 3 and Table 4). The BODEX index is divided into quartiles: quartile 1 (0–2 points), quartile 2 (3–4 points), quartile 3 (5–6 points) and quartile 4 (7–9 points), with a maximum BODEX value of 9 points. The median BODEX index score was 2.91, 95% CI [2.62, 3.21] (Figure 3). A Kaplan–Meier curve plot considering BODEX index values divided by quartiles showed that patients in the fourth quartile had a worse prognosis than those in the first quartile (HR 7.93, 95% CI [2.63, 23.86], *p* < 0.001) (Figure 5). As the BODEX does not take into account the results of the 6MWD, it is well suited for use in real-world clinical practice. However, this index takes into account the frequency of COPD exacerbations, which cannot always be documented in real clinical practice. In the present study, we evaluated the progression of COPD over the three years between the first and second control points to calculate the average frequency of exacerbations per year.

The e-BODE index takes into account body mass index (B), degree of airflow obstruction (O), severity of dyspnoea on the mMRC scale (D) and exercise capacity (E), as assessed by the six-minute walk test and exacerbation frequency (EX). The e-BODE index can reach a score of 12 and is divided into quartiles: quartile 1 (0–2 scores), quartile 2 (3–4 scores), quartile 3 (5–6 scores) and quartile 4 (7–12 scores). The median e-BODE index score was 3.67, 95% CI [3.22, 4.12] (Figure 3). A Kaplan–Meier curve plot considering e-BODE index values divided into quartiles showed that patients with the fourth quartile had a worse prognosis compared to the first quartile (HR 6.11, 95% CI [3.32, 11.23], *p* < 0.001) (Figure 5, Table 3 and Table 4). In addition, the simultaneous consideration of body mass index and exacerbation frequency increased the prognostic significance of the BODEX index compared to BODE and BODEX (Table 3 and Table 4).

**Figure 6:**
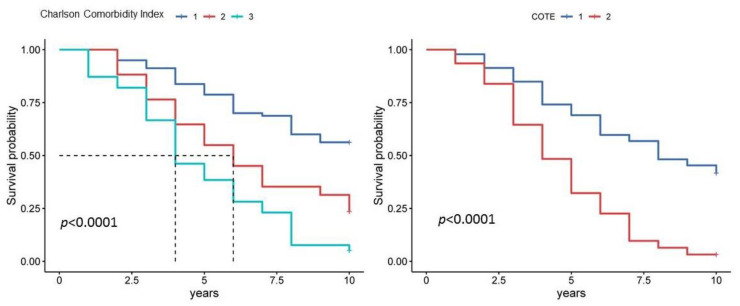
Kaplan–Meier curves for Charlson Comorbidity Index and COTE index in 10-year survival estimates. Notes: For the Charlson Comorbidity Index, subgroup 1 is a score of 1 to 4, subgroup 2 is a score of 5 to 6, and subgroup 3 is a score of 7 or higher. For the COTE index, subgroup 1 is a score of 0 to 2 and subgroup 2 is a score of 3 or higher.

Comorbidity, as assessed by the Charlson Comorbidity Index, was also associated with prognosis in COPD. An increased Charlson Comorbidity Index was associated with a worse prognosis for both 5-year and 10-year patient survival (Figure 6, Table 3 and Table 4). A Kaplan–Meier curve plot of the Charlson Comorbidity Index divided into three subgroups showed that patients in the third subgroup had a worse prognosis compared to the first subgroup (HR 3.82, 95% CI [2.25, 6.49], *p* < 0.001) (Figure 6).

The COTE index, which takes into account comorbidities relevant to COPD, showed a higher prognostic significance compared to the Charlson Comorbidity Index (Figure 6, Table 3 and Table 4). The Kaplan–Meier curve plot, which considered COTE index scores divided into two subgroups, showed that patients in the second subgroup (sum score above 3) had a worse prognosis compared to the first subgroup (HR 5.66, 95% CI [3.03, 10.55], *p* < 0.001) (Figure 6).

**Figure 7:**
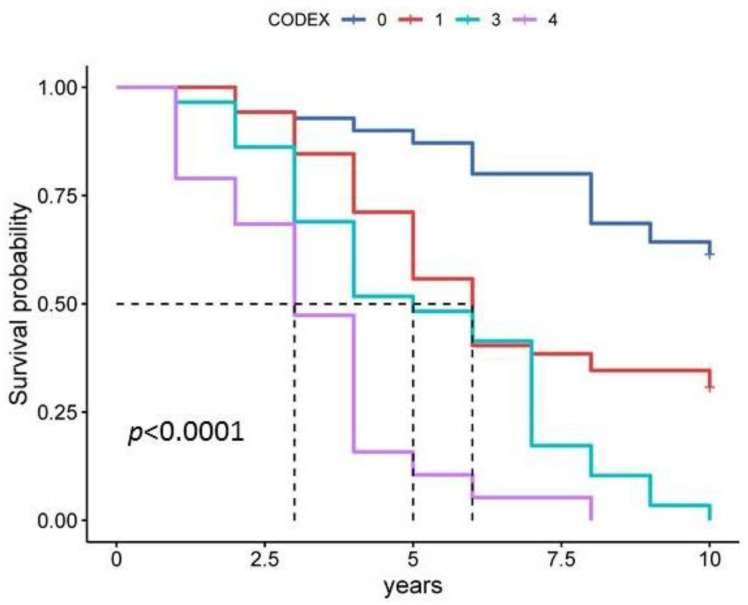
Kaplan–Meier curve plot for the CODEX index in estimating 10-year survival. Note: Quartile 1 is a score of 0 to 2, quartile 2 is a score of 3 to 4, quartile 3 is a score of 5 to 6 and quartile 4 is a score of 7 to 10.

The CODEX index includes comorbidity data based on the Charlson Comorbidity Index (C), degree of airflow obstruction (O), severity of dyspnoea on the mMRC scale (D) and frequency of exacerbations (EX). Index scores ranged from 0 to 10, with higher scores indicating a higher risk of death. The median CODEX index score was 3.51, 95% CI [3.17, 3.86] (Figure 3). The CODEX index had the highest prognostic significance for both 5-year and 10-year survival (Figure 7, Table 3 and Table 4). A Kaplan–Meier curve plot considering CODEX index values divided into quartiles showed that patients in the fourth quartile had a worse prognosis compared to the first quartile (HR 8.23, 95% CI [3.33, 20.37], *p* < 0.001) (Figure 7). These data highlight the importance of a comprehensive assessment of different factors relevant to the heterogeneous course of COPD.

The data in Table 3 and Table 4 show that all the indices considered have different prognostic significance for 5-year and 10-year survival. An analysis of the correlations of the indices with the number of years lived from the second control point showed that the highest values were for the CODEX, eBODE and BODEX indices, which include data on the frequency of COPD exacerbations (Table 5). These data are consistent with the known evidence that infectious exacerbations of COPD contribute not only to local, but also to systemic inflammation, which may act as a link to some comorbid conditions and affect prognosis [14].

This study used a Cox proportional hazards regression analysis to evaluate the prognostic significance of the BODE, eBODE, BODEX, CODEX, COTE, ADO and Charlson Comorbidity Index in relation to the 10-year survival rate of patients diagnosed with COPD. The results of the Cox proportional hazards regression model indicated that an increase in each index score was associated with a higher risk of mortality. The results presented in Table 6 show that all six multidimensional indices and the Charlson Comorbidity Index were significantly associated with an increased risk of mortality in COPD patients with *p*-values less than 0.0001 and HRs greater than 1. The hazard ratios ranged from 1.3031 (95% CI 1.2179–1.3942) for the BODE index to 1.5514 (95% CI 1.3693–1.7578) for the COTE index. The different number of maximum possible points in each index should be taken into account.

Therefore, the results of this study suggest that the BODE, eBODE, BODEX, COTE, CODEX, ADO and Charlson Comorbidity Index can be useful prognostic tools for assessing the risk of mortality in COPD patients.

**Figure 8:**
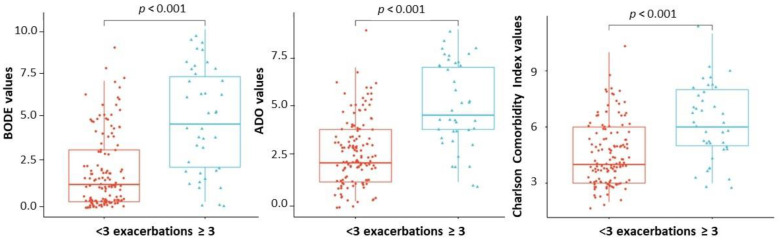
Jitter plot values of the BODE, ADO and Charlson Comorbidity Index in patients with an exacerbation frequency of up to three and more than three per year.

The inclusion of exacerbation frequency in the index has been shown to increase its prognostic value. Therefore, a comparative analysis of the BODE, ADO and Charlson Comorbidity Index, which do not take exacerbation frequency into account, was performed in patients with an exacerbation frequency of up to three exacerbations per year and in those with an exacerbation frequency of more than three per year (Figure 8).

As shown in Figure 8, statistically significant differences were found in the values of the BODE, ADO and Charlson Comorbidity Index in patients with an exacerbation frequency of up to three and more than three per year.

**Figure 9:**
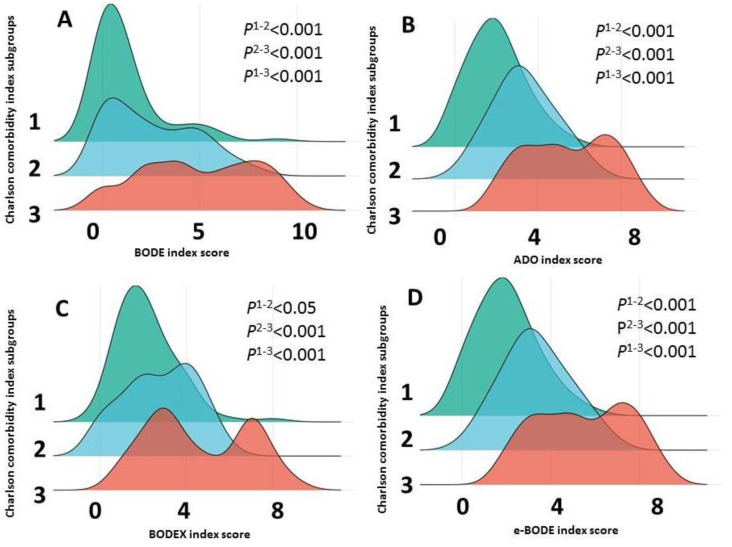
Plot of BODE (**A**), ADO (**B**), BODEX (**C**) and e-BODE (**D**) as a function of the severity of comorbid disease, as assessed by the Charlson Comorbidity Index and classified into 3 subgroups. Note: Statistical significance of differences in BODE, ADO, BODEX, and e-BODE index values in patients divided into three subgroups according to Charlson Comorbidity Index values are shown.

Given the importance of comorbidity in the prognosis of COPD, its association with the multidimensional indices was analysed. The Charlson Comorbidity Index, divided into three subgroups (subgroup 1 is a score of 1 to 4, subgroup 2 is a score of 5 to 6 and subgroup 3 is a score of 7 or more), showed an association with increasing BODE, ADO, BODEX and e-BODE (Figure 9).

The analysis showed that indicators such as dyspnoea, airflow obstruction, comorbidity and exacerbation frequency made the most significant contribution to the prognosis of COPD.

## 4. Discussion

The present study analysed the prognostic significance of multidimensional indices including various factors reflecting the clinical and functional characteristics of COPD. Data were obtained by analysing the course of COPD in 170 male patients examined at baseline and after three years (first and second control points). At 5 and 10 years after the second control point, patient survival was assessed, and factors associated with prognosis were analysed. Demographic and clinical data were analysed, including the presence of symptoms, comorbidities and spirometry data. Clinical characteristics were assessed using multidimensional indices proposed to assess the course and prognosis of COPD. The prognostic significance of the BODE, eBODE, BODEX, CODEX, ADO and Charlson Comorbidity Index were analysed.

The assessment of markers of COPD progression and prognosis is of great clinical interest [15,16,17,18]. It should be noted that COPD is a disease with pulmonary and extrapulmonary clinical heterogeneity, which is reflected in the variability of clinical features such as the severity of symptoms, the dynamics of pulmonary function decline and the presence and nature of comorbidities [6,19]. The clinical heterogeneity of COPD phenotypes does not allow the development of a single tool for predicting disease progression and clinical outcomes; therefore, multidimensional indices assessing different clinical features of the disease have been developed. Multidimensional indices have been shown to be better predictors of survival than any of the individual indices [9,20]. Multidimensional indices have been found to have good prognostic significance and can be used to assess the course and survival of COPD in long-term follow-up. Several indices are widely accepted, such as the BODE index and its modifications that include exacerbation frequency scores (BODEX and eBODE), CODEX, ADO, as well as tools to assess prognostically significant comorbidity, such as the Charlson Comorbidity Index and COTE. They differ in the composition of variables analysed, but all incorporate available clinical data and are easy to use in real clinical practice.

The decline in lung function is an important feature of the progressive course of COPD [21,22]. Despite the diagnostic value of FEV1, this parameter does not have high prognostic significance and correlates poorly with clinical features of COPD such as symptom severity, quality of life, exacerbation frequency and exercise intolerance [7,23,24]. On the other hand, FEV1 is used as part of most multidimensional indices. Dyspnoea is an important multidimensional clinical symptom that has been the subject of numerous studies [25,26]. Although dyspnoea is a subjective symptom and does not correlate well with lung function, it is widely used in multidimensional indices [27].

The presence of comorbidities is an important feature of the clinical heterogeneity of COPD [28]. Comorbid conditions, such as atherosclerotic cardiovascular disease, share some common risk factors and pathogenesis and contribute significantly to the prognosis of COPD [29]. In this regard, indices that take into account comorbidity, including CODEX, have good prognostic significance. This indicates the need to consider comorbidities when assessing the course of COPD. 

The Charlson Comorbidity Index is one of the most common and widely used indices to assess the risk of death from comorbidities in longitudinal studies [30]. It is a scoring system that assesses age and the presence of certain comorbidities of various organs and systems, each of which is assigned a score. As the Charlson Comorbidity Index primarily predicts 10-year survival, it is of interest for estimating life expectancy and can be used to plan long-term management.

In addition, Divo et al. developed the COPD comorbidity test (COTE) index, which takes into account comorbidities that affect survival in patients with COPD [31].

COPD exacerbations are an important clinical feature of the disease, influencing disease progression and prognosis [32,33]. Multidimensional indices that take into account the frequency of exacerbations have shown good prognostic significance. The CODEX index has been shown to be the most useful in predicting short- and medium-term survival in patients hospitalised for acute exacerbations of COPD compared with other multidimensional COPD indices [12].

Thus, the main conclusion of this study is that multidimensional indices including several physiological and clinical parameters, such as age, dyspnoea, exacerbation rate, exercise capacity and comorbidity, in addition to FEV1, may be useful for assessing prognosis in patients with COPD.

### Limitations of the Study

It should be noted that this study had some limitations, such as a small sample size. In addition, only male patients were analysed for the multidimensional indices. On the other hand, this limitation made it possible to exclude the influence of sex-specific characteristics in the course of COPD, in the perception and interpretation of symptoms, and in the structure of comorbid pathology. A considerable amount of evidence suggests that COPD in women may be associated with some features of the course of the disease that lead to adverse outcomes, thus increasing attention to the problem of COPD in this group. The findings could be used to plan further studies that take these limitations and findings into account. Other promising directions for future research could be to assess the prognostic value of multidimensional indices in different COPD phenotypes and to consider some biochemical markers in assessing the prognosis of COPD in long-term follow-up.

## 5. Conclusions

The results suggest that multidimensional indices that take into account exacerbation frequency, airflow obstruction, dyspnoea and the presence of comorbid conditions are good tools for assessing the course and prognosis of COPD.

## Figures and Tables

**Table 1:** Clinical characteristics of patients.

Characteristics	Data
Age	60.02 (95% CI 58.68; 61.34)
Body mass index (BMI)	26.6 (95% CI 26.13; 27.07)
Pack-year index	37.72 (95% CI 36.41; 39.03)
FEV1, %	72.05 (95% CI 69.94; 74.17)
Dyspnoea, MRC (2 time points)	1.56 (95% CI 1.37; 1.75)
Frequency of COPD exacerbations per year	1.94 (95% CI 1.77; 2.1)
GOLD 1 (2 time points)	14.7% (25)
GOLD 2 (2 time points)	64.7% (110)
GOLD 3 (2 time points)	16.47% (28)
GOLD 4 (2 time points)	4.11% (7)

**Table 2:** Correlation analysis with COPD stage at the second control point.

Parameter	r (95% CI)	*p*
BODEX index	0.7270 (0.6473; 0.7909)	<0.0001
eBODE index	0.6735 (0.5820; 0.7482)	<0.0001
CODEX index	0.6657 (0.5725; 0.7419)	<0.0001
BODE index	0.6618 (0.5678; 0.7387)	<0.0001
ADO index	0.6129 (0.5094; 0.6990)	<0.0001
Charlson Comorbidity Index	0.2932 (0.1493; 0.4249)	=0.0001
COTE Index	0.1941 (0.0448; 0.3348)	=0.0112

**Table 3:** Results of receiver operating characteristic (ROC) curve analysis for 5-year survival.

Parameter	AUC (95% CI)	*p*	Sensitivity (%)	Specificity (%)
CODEX index	0.795 (0.726; 0.853)	<0.0001	85.94%	57.55%
ADO index	0.791 (0.722; 0.849)	<0.0001	84.37%	58.43%
eBODE index	0.787 (0.717; 0.846)	<0.0001	87.50%	69.81%
BODEX index	0.779 (0.709; 0.839)	<0.0001	75.00%	66.04%
BODE index	0.771 (0.701; 0.832)	<0.0001	82.81%	70.75%
COTE index	0.725 (0.652; 0.791)	<0.0001	73.44%	64.15%
Charlson Comorbidity Index	0.709 (0.635; 0.776)	<0.0001	73.44%	59.43%

Abbreviations: CI: confidence interval, AUC: area under the receiver operating characteristic curve.

**Table 4:** Results of receiver operating characteristic (ROC) curve analysis for 10-year survival.

Parameter	AUC (95% CI)	*p*	Sensitivity (%)	Specificity (%)
CODEX index	0.847 (0.784; 0.898)	<0.0001	63.96%	94.92%
eBODE index	0.830 (0.765; 0.883)	<0.0001	73.87%	89.83%
ADO index	0.826 (0.760; 0.880)	<0.0001	54.95%	96.61%
BODEX index	0.822 (0.756; 0.877)	<0.0001	66.67%	83.05%
BODE index	0.809 (0.741; 0.865)	<0.0001	70.27%	89.83%
COTE index	0.785 (0.715; 0.844)	<0.0001	66.67%	81.36%
Charlson Comorbidity Index	0.773 (0.702; 0.833)	<0.0001	68.47%	76.27%

Abbreviations: CI: confidence interval, AUC: area under the receiver operating characteristic curve.

**Table 5:** Correlation analysis of multidimensional index scores with years lived since baseline.

Parameter	r (95% CI)	*p*
CODEX index	−0.6097 (−0.6963; −0.5055)	<0.0001
eBODE index	−0.5791 (−0.6711; −0.46895)	<0.0001
BODEX index	−0.5779 (−0.6701; −0.4681)	<0.0001
ADO index	−0.5651 (−0.6596; −0.4532)	<0.0001
BODE index	−0.5477 (−0.6451; −0.4329)	<0.0001
COTE index	−0.4894 (−0.5960; −0.3658)	<0.0001
Charlson Comorbidity Index	−0.4688 (−0.5785; −0.3425)	<0.0001

Abbreviations: CI: confidence interval.

**Table 6:** Ten-year survival hazard ratios for multidimensional indices in the Cox regression model.

Parameter	HR (95% CI)	*p*
COTE Index	1.5514 (1.3693; 1.7578)	<0.0001
BODEX Index	1.4871 (1.3521; 1.6357)	<0.0001
CODEX index	1.4274 (1.3164; 1.5478)	<0.0001
ADO Index	1.4139 (1.2951; 1.5437)	<0.0001
BODE Index	1.3031 (1.2179; 1.3942)	<0.0001
eBODE Index	1.2932 (1.2155; 1.3759)	<0.0001
Charlson Comorbidity Index	1.3512 (1.2291; 1.4855)	<0.0001

Abbreviations: CI: confidence interval, HR: hazard ratio.

## Data Availability

Data can be provided upon request to the author by e-mail.
